# Re-evaluating a single-arm trial of favipiravir for severe fever with thrombocytopenia syndrome in Japan: A proposal to use real-world data as external controls

**DOI:** 10.1371/journal.pntd.0013279

**Published:** 2025-08-01

**Authors:** Kenji Kubo, Mayumi Toyama, Ken-ichiro Kobayashi, Nobuhiro Komiya, Takeo Nakayama

**Affiliations:** 1 Department of Emergency Medicine and Department of Infectious Diseases, Japanese Red Cross Wakayama Medical Centre, Wakayama, Japan; 2 Department of Health Informatics, Kyoto University Graduate School of Medicine and Public Health, Kyoto, Japan; 3 Department of Infectious Diseases, Japanese Red Cross Wakayama Medical Centre, Wakayama, Japan; Beijing Children's Hospital Capital Medical University, CHINA

Severe fever with thrombocytopenia syndrome (SFTS) is an emerging viral haemorrhagic fever that has been increasingly reported in Asia, particularly in China and Japan [1]. Suemori et al. reported the potential efficacy of favipiravir therapy for patients with SFTS, and their contribution to the field is commendable [2]. Subsequently, favipiravir was approved in Japan for the treatment of SFTS in June 2024. Although this study was not a randomised controlled trial, the authors’ decision to use a single-arm design is justifiable, considering that SFTS is a potentially fatal emerging infectious disease for which only a limited number of cases are available for study.

Suemori et al. reported a 17% mortality in the favipiravir-treated group, concluding that “favipiravir treatment may have reduced the case fatality rate of patients with SFTS by approximately 10% compared to those reported so far in epidemiological surveys in Japan [2].” However, is this estimated effect size of a ‘10% reduction’ appropriate?

As the authors indicated, comparison with external controls is crucial in single-arm trials [2]. Establishing appropriate external controls is recommended for such studies. Recently, a new paradigm has emerged that utilises various real-world data (RWD) to address this challenge [3]. Burcu et al. and Lambert et al. recommend a structured approach for using external controls in single-arm studies [4–6], which involves: 1) defining the estimand, including the target population and effect measure; 2) selecting external controls that match baseline characteristics; and 3) adjusting for confounding using statistical methods. Potential sources of external controls include observational cohorts, registries, electronic health records, claims data, and prescription data. In exceptional cases, when the natural history of a disease is well-defined and the treatment effect is markedly superior to historical controls, single-arm trials without external comparators may still provide robust evidence [3,6,7].

The recent publication of several Japanese studies using large-scale RWD on SFTS [8,9] led us to examine their potential application as external controls. These studies, along with the two external controls adopted by Suemori et al. [10,11], are summarised in Table 1.

**Table 1:** Comparison of Japanese studies on severe fever with thrombocytopenia syndrome.

Study (author, published year)	Suemori, 2021	External controls used for comparison by Suemori et al.		External controls newly included in this study			
		Kato, 2016	Kobayashi, 2020	Kutsuna, 2023		Kutsuna, 2024	
Study Characteristics							
Study design	Prospective, single-arm trial	Retrospective observational	Retrospective observational	Retrospective observational		Retrospective observational	
Study period, years	2016–18	2013–14	2013–17	2013–21		2013–21	
SFTS definition	PCR-confirmed	PCR-confirmed	PCR-confirmed	ICD-10 code A938-based*		ICD-10 code A938-based*	
Favipiravir use	Yes	No	No	No		No	
Mortality definition	28-day mortality	Unspecified timing	Unspecified timing	In-hospital mortality		In-hospital mortality	
Patient Characteristics							
Patients, n	23	49 (of 96 invited)	133 (of 303 invited)	412		425	
				steroid pulse	non-pulse	antibiotic group	non-antibiotic
				66	346	287	138
Age, median (interquartile range)	71.0 (65, 81)	78 (65, 84)	73 (65,82)	75 (68, 80)	73 (65, 81)	73 (65,80)	73 (65,80)
Male, %	61	35	47	64	49	52	52
Charlson comorbidity index, %	NR	NR	NR	0: 62, 1: 24, 2: 9, ≥ 3: 5	0: 70, 1: 17, 2: 10, ≥ 3: 3	0: 68, 1: 20, 2:8, ≥ 3: 4	0: 71, 1: 16, 2: 11, ≥ 3: 2
Hypertension, %	52	47	35	NR		NR	
Malignancy, %	4	4	7	NR		NR	
Diabetes mellitus, %	17	24	20	NR		NR	
Intensive care, %	26 (mechanical ventilation)	29	NR	41	27	35	13
Time from onset, median days (interquartile range)	To favipiravir initiation, 4 (3, 5)	To admission, 4 (2–5)	To initial hospital visit, 3 (2–5)	NR		NR	
Mortality, %	17.3	31	27	17.1		18.8	

SFTS = Severe fever with thrombocytopenia syndrome, ICD-10 = the International Classification of Diseases, Tenth Revision, NR = Not reported. * In Japan, SFTS is generally diagnosed by PCR at regional health laboratories, and registrations in the DPC database are considered to be based on PCR confirmation.

First, when defining the target population, it is crucial to account for differences between the Japanese and Chinese strains of the SFTS virus. Evidence suggests that severity and case fatality rates may vary between these strains. Since the study by Suemori et al. focuses on SFTS cases in Japan, it is appropriate to use studies involving Japanese SFTS cases as external controls [12]. In Japan, SFTS is diagnosed by PCR at local health centres, and, since nationwide surveillance began in 2013, reporting of all laboratory-confirmed cases has been mandatory [1]. Therefore, reported SFTS cases in Japan are considered laboratory-confirmed (Table 1) [8–11].

When assessing the suitability of the target population for external control, all four studies are considered eligible, as they were conducted prior to the approval of favipiravir for SFTS [8–11]. Given that the study period of Suemori et al. was 2016–2018, the studies by Kutsuna et al., which cover this timeframe, constitute more appropriate external controls than those by Kato et al. or Kobayashi et al. Kutsuna et al. (2023; 2024) used the Diagnosis Procedure Combination (DPC) database, a nationwide administrative claims system covering inpatient admissions from more than 1,200 acute care hospitals in Japan [8,9]. These studies encompassed approximately 70% of all reported SFTS cases between 2013 and 2021 (412/587 and 425/587), underscoring the representativeness of this RWD source. An important limitation is that no information is available on the time elapsed since symptom onset. In contrast, Kato et al. and Kobayashi et al. conducted retrospective questionnaire-based studies of treating physicians to obtain clinical characteristics and outcomes [10,11]. The response rates were approximately 40–50% (49/96, 51% for Kato; 133/303, 44% for Kobayashi), raising concerns about non-response bias. Regarding mortality outcomes, Kutsuna et al. reported short-term mortality, aligning with the methodology of Suemori et al [2,8,9]. In contrast, Kato et al. and Kobayashi et al. did not prespecify the timing of mortality assessments, as the outcomes were collected only retrospectively through physician questionnaires [10,11].

Based on the above, the RWD used by Kutsuna et al. represent a more suitable candidate for an external control than the studies by Kato et al. and Kobayashi et al., which were cited by Suemori et al. According to Kutsuna et al., the mortality in the non-favipiravir group was 17.1% and 18.8%, which did not differ substantially from the 17.3% observed in the favipiravir-treated group in the study by Suemori et al [2,8,9]. However, while the inclusion criteria in the studies by Suemori et al. and Kutsuna et al. appear broadly similar, a key difference in exclusion criteria warrants attention: Suemori et al. excluded patients who presented > 7 days after symptom onset, whereas Kutsuna et al. excluded those discharged ≤ 3 days after admission. This methodological discrepancy introduces a high risk of selection bias when comparing these cohorts.

Next, to estimate treatment effects appropriately, matching and statistical adjustment are generally required—an approach emphasised in articles by Burcu et al., Lambert et al., as well as in regulatory guidance [4–6]. However, statistical analysis was deemed infeasible because the comparison was conducted post hoc, individual-level data were unavailable, and the sample size in the Suemori et al. study was very small. A comparison of characteristics across external control studies, as summarised in Table 1, reveals that they are relatively similar, with median ages in their 70s [8–11]. Although several variables could not be assessed owing to the absence of individual-level data, the prevalence of underlying conditions such as diabetes and malignancy was relatively low, and severe cases accounted for approximately 10–20%. Off-label antiviral agents (e.g., ribavirin) were excluded from the Suemori et al. study, and no such treatments were reported in the other studies.

For additional context, the mortality reported in studies on SFTS in China was as follows: in the randomised controlled trial by Li et al., the control group had a mortality of 18.3%, while the favipiravir-treated group had a mortality of 9.5% (odds ratio, 0.47; 95% confidence interval, 0.17–1.25) [13]. Similarly, in the observational study by Yuan et al. using propensity-score matching, the control group had a mortality of 20.0%, while the favipiravir-treated group had a mortality of 9.0% (odds ratio, 0.38; 95% confidence interval, 0.23–0.65) [14]. The median ages of the control groups were 62.4 and 63 years, respectively, notably younger than those in the Japanese studies. Additionally, these studies did not report key severity indicators, such as ICU admission. Although these findings suggest favipiravir’s potential benefit, differences in epidemiological factors hinder direct comparisons with Japanese data, resulting in uncertainty regarding its effectiveness in Japanese SFTS cases.

Furthermore, since favipiravir is a relatively new medication, its potential adverse effects have not been fully explored. Notably, studies evaluating favipiravir for COVID-19 have demonstrated no clinical benefit and have reported an increase in adverse events [15]. In clinical decision-making, weighing the potential benefits of reduced mortality against the risks of adverse events is essential. Overestimating the expected benefits of the drug could lead to inappropriate treatment decisions.

In conclusion, the single-arm study by Suemori et al. does not allow for a precise estimation of the effect size of mortality reduction. Therefore, clinicians should interpret its findings with caution, incorporating the considerations outlined above before reaching clinical decisions. Building on recent regulatory guidance to address the inherent limitations of single-arm trials [5,6], we propose a hybrid design that integrates a prospective single-arm study with a real-world external control cohort. The prospective component, as exemplified by Suemori et al., enables systematic collection of key clinical data, including time from symptom onset, virological and laboratory variables, and pharmacokinetic and pharmacodynamic parameters [2]. Concurrently, DPC-based studies such as those by Kutsuna et al. demonstrate the feasibility of assembling sufficiently large external control groups, even for rare diseases [8,9]. Ensuring methodological rigour in such hybrid designs necessitates prespecified eligibility criteria, robust matching strategies, appropriate sample-size calculations, and standardised data capture. Future research should quantify the real-world effectiveness of favipiravir in reducing SFTS-related mortality in Japan, and determine whether its potential benefits outweigh the risk of adverse events.
